# Effects of Nano-SiO_2_ on the Pore Structure and Crack Behavior of Basalt Fiber-Reinforced Coal Gangue–Slag Geopolymer Concrete

**DOI:** 10.3390/ma19153229

**Published:** 2026-07-29

**Authors:** Weizi Wang, Lianyong Zhu, Tao Li, Renfei Gao

**Affiliations:** School of Water Conservancy and Architectural Engineering, Tarim University, Alar 843300, China; wangweizi1224@163.com (W.W.); 15213905080@163.com (T.L.); 15588733729@163.com (R.G.)

**Keywords:** coal gangue–slag geopolymer concrete, nano-SiO_2_, digital image correlation, crack evolution, mechanical properties

## Abstract

To improve the mechanical performance and crack resistance of coal gangue–slag geopolymer concrete, basalt fiber-reinforced geopolymer concrete was prepared using calcined coal gangue powder and slag as composite precursors. The effects of nano-SiO_2_ dosage on strength, water absorption, and crack evolution under splitting tensile loading were investigated. Digital image correlation (DIC), scanning electron microscopy (SEM), and mercury intrusion porosimetry (MIP) were further employed to characterize the crack evolution and microstructural changes associated with nano-SiO_2_ incorporation. The results show that the performance of the specimens was strongly dependent on the nano-SiO_2_ dosage, with the NS0.5 mixture (0.5% nano-SiO_2_) exhibiting the most pronounced improvement. Compared with the reference mixture, the 28 d compressive strength and splitting tensile strength of NS0.5 reached 86.80 MPa and 6.02 MPa, corresponding to increases of 17.67% and 39.97%, respectively; meanwhile, the 24 h water absorption decreased from 4.78% to 4.25%. The DIC results indicate that 0.5% nano-SiO_2_ delayed the localization of maximum principal strain and the penetration of the main crack, reducing the peak crack width from 0.487 mm to 0.222 mm, with a reduction of 54.4%. The microstructural results show that an appropriate nano-SiO_2_ dosage reduced the total porosity from 13.93% to 5.03%, shifted the pore structure from macropore-dominated to fine-pore-dominated, and decreased the proportion of coarse connected pores and crack-like pores. In contrast, the reduced enhancement observed at higher nano-SiO_2_ dosages may be associated with poorer nanoparticle dispersion and increased local heterogeneity, although particle agglomeration was not directly verified in this study. Overall, the improvements obtained with 0.5% nano-SiO_2_ are directly consistent with matrix densification and pore-structure refinement. Micro-filling, heterogeneous nucleation, and additional gel formation are proposed as plausible contributing mechanisms rather than directly verified processes.

## 1. Introduction

Ordinary Portland cement concrete has long been the dominant structural material in civil engineering. However, cement production is associated with high energy consumption and substantial carbon emissions, while the stockpiling of large quantities of industrial solid wastes imposes considerable pressure on ecosystems and land resources. Therefore, the development of low-carbon binders using industrial solid wastes as primary raw materials has become an important research direction in concrete materials [1,2,3,4]. Geopolymer materials are generally produced from solid precursors rich in Si and Al, which undergo alkali activation to form binding systems mainly composed of aluminosilicate gels and calcium aluminosilicate hydrate gels. These materials are characterized by broad raw-material availability, high solid-waste utilization potential, and adjustable mechanical performance [5,6,7].

Coal gangue is a bulk solid waste generated during coal mining and washing, and is mainly composed of aluminosilicate components such as SiO_2_ and Al_2_O_3_ [8,9]. After appropriate thermal activation, inert or low-reactivity mineral phases in coal gangue, such as kaolinite, can undergo dehydroxylation and partially transform into highly reactive amorphous aluminosilicate phases, thereby enabling their participation as geopolymer precursors in alkali-activated reactions [9,10,11,12]. Slag is rich in CaO, SiO_2_, and Al_2_O_3_ and possesses high latent hydraulic activity. Under alkaline conditions, it can dissolve and promote the formation of C–A–S–H gel or C–(N)–A–S–H composite gel [13,14]. The combined use of calcined coal gangue and slag not only improves the resource utilization of coal gangue, but also enhances strength development in the geopolymer matrix through the synergistic contribution of low-calcium aluminosilicate reactions and calcium-rich gel formation [15,16,17]. Nevertheless, coal gangue–slag geopolymer concrete may still suffer from relatively high pore connectivity, weak interfacial transition zones, and pronounced tensile brittleness, which can lead to rapid crack propagation and penetration under tensile or splitting loads [16,18,19].

Fiber reinforcement is an effective approach for improving brittle failure and crack propagation behavior in geopolymer-based materials. Previous studies have shown that basalt fibers possess high tensile strength, high elastic modulus, good corrosion resistance, and favorable durability, giving them strong reinforcement potential in cement-based and geopolymer composites [20,21]. Ranjbar and Zhang [22] reported that the incorporation of fibers can improve the toughness, ductility, and crack-control capacity of geopolymers through crack bridging, fiber pull-out, and interfacial frictional energy dissipation. Studies by Dias and Thaumaturgo [23], Li and Xu [24], and Wang et al. [25,26] further demonstrated that basalt fibers can enhance the tensile strength, fracture toughness, and fracture energy of geopolymer concrete, improve its impact resistance and elastic modulus, and effectively suppress crack propagation and crack opening displacement. However, the reinforcing effect of fibers is governed not only by their intrinsic properties, but also by the compactness of the matrix, pore structure, and fiber–matrix interfacial bonding. Ranjbar et al. [27] and Yazid et al. [28] indicated that strong interfacial bonding is essential for effective stress transfer and fiber toughening. When the matrix contains abundant pores, microcracks, or weak interfaces, fibers are prone to debonding and pull-out, thereby reducing their reinforcing efficiency. Therefore, fiber reinforcement alone is insufficient to fundamentally suppress early-stage localization of tensile damage, and further optimization of the matrix microstructure and interfacial characteristics is required. Assaedi et al. [29] and Alomayri et al. [30] found that nano-SiO_2_ can improve the mechanical properties and interfacial bonding of fiber-reinforced geopolymer composites by filling pores, promoting gel formation, and densifying the microstructure. Accordingly, basalt fibers mainly enhance crack resistance through crack bridging and energy dissipation, whereas nano-SiO_2_ improves fiber reinforcement efficiency by modifying the pore structure and interfacial properties of the matrix. Their combined use is therefore expected to synergistically improve the strength, toughness, and crack-control capacity of coal gangue–slag geopolymer concrete.

Nano-SiO_2_ has been widely used for microstructural modification of cement-based and geopolymer materials because of its small particle size, high specific surface area, and strong surface activity. Aggarwal et al. [31] and Rashad [32] concluded that nano-SiO_2_ can improve hydration or geopolymerization processes through filling, pozzolanic, and nucleation effects, thereby enhancing the mechanical properties and durability of cementitious materials. Wang et al. [33] found that nano-SiO_2_ promotes hydration and C–S–H gel formation in ordinary Portland cement systems while refining the pore structure. For geopolymer systems, Phoo-Ngernkham et al. [34] reported that nano-SiO_2_ can shorten the setting time and increase the compressive strength of high-calcium fly ash geopolymers, which was attributed to C–S–H-like gel formation and matrix densification. Gao et al. [35] showed that nano-SiO_2_ affects reaction kinetics, gel structure, porosity, and strength development in alkali-activated slag–fly ash blends. Deb et al. [36] further demonstrated that an appropriate nano-SiO_2_ dosage can promote the formation of C–S–H/C–A–S–H and N–A–S–H reaction products in fly ash, fly ash–slag, and fly ash–cement geopolymer systems, resulting in a denser matrix. Deb et al. [37] also found that nano-SiO_2_ can reduce water absorption and porosity and improve the acid resistance of geopolymer mortars. Wang et al. [38] similarly reported that nano-SiO_2_ can regulate hydration reactions and microstructural pore characteristics in alkali-activated slag systems, thereby improving matrix compactness. However, the enhancement effect of nano-SiO_2_ does not increase linearly with dosage. Rong et al. [39] noted that excessive nano-SiO_2_ can reduce reinforcement efficiency due to particle agglomeration, while Kong et al. [40] showed that nano-SiO_2_ agglomeration significantly affects the fresh properties and dispersion state of cementitious pastes. Deb et al. [36] also observed that when the nano-SiO_2_ dosage exceeds an appropriate range, the strength of geopolymer systems may decrease and the microstructure may become looser. Therefore, the optimum dosage of nano-SiO_2_ in coal gangue–slag geopolymer concrete and its influence on crack evolution and pore structure require further clarification.

Existing studies on nano-modified geopolymer concrete have mainly focused on compressive strength, flexural strength, durability, pore structure, or microstructural morphology [31,32,33,34,35,36,37,38]. Although Zhang et al. [41] investigated the fracture parameters of PVA fiber- and nano-SiO_2_-modified geopolymer/alkali-activated mortars, their evaluation was still mainly based on conventional mechanical indices and discrete parameters such as crack mouth opening displacement, making it difficult to fully characterize the complete process of crack initiation, strain localization, and crack opening evolution under tensile failure. Traditional mechanical tests generally provide only peak strength, load–displacement response, and final failure morphology, and are insufficient for revealing the dynamic evolution of cracks from localized damage and stable propagation to unstable penetration. Digital image correlation (DIC) is a non-contact full-field deformation measurement technique that continuously obtains surface displacement and strain fields through grayscale image correlation analysis [42,43]. Alam et al. [44] used DIC to monitor crack opening in concrete and demonstrated its applicability for quantifying crack width and size effects. Fayyad and Lees [45] further showed that DIC can capture crack propagation and branching in reinforced concrete beams. For geopolymer systems, Xie et al. [46] used DIC to analyze the fracture behavior of geopolymer concrete and showed that this technique can reveal crack propagation paths and the evolution of the fracture process zone. Tang et al. [47] applied DIC to investigate the failure mechanism of geopolymeric recycled concrete and found good agreement between DIC-identified strain concentration zones and macroscopic crack locations. Gehri et al. [48] proposed a DIC-based method for automated crack detection and crack kinematics measurement, enabling further quantification of crack width and slip. Mata-Falcón et al. [49] indicated that the combined use of DIC and other distributed measurement techniques can continuously characterize deformation, cracking, and load-bearing behavior in concrete structures. Thus, integrating DIC with SEM and MIP can provide multiscale evidence for interpreting the effects of nano-SiO_2_ in terms of mechanical response, crack evolution, and pore-structure characteristics.

Based on the above considerations, this study prepared basalt fiber-reinforced coal gangue–slag geopolymer concrete using calcined coal gangue powder and slag as composite precursors, with nano-SiO_2_ dosage as the main variable. The effects of nano-SiO_2_ on compressive strength, splitting tensile strength, and water absorption were systematically investigated. DIC was then employed to obtain the maximum principal strain field and main crack opening displacement during splitting tensile loading, enabling analysis of damage localization and crack propagation under different nano-SiO_2_ dosages. Furthermore, SEM and MIP were used to reveal the effects of nano-SiO_2_ on matrix microstructure, pore-size distribution, and crack-control capacity. This study aims to clarify the regulatory effect of nano-SiO_2_ on the mechanical properties and crack evolution behavior of basalt fiber-reinforced coal gangue–slag geopolymer concrete, providing a basis for mixture optimization and crack-resistant design of high-performance industrial solid-waste-based geopolymer concrete.

## 2. Experimental Section

### 2.1. Experimental Materials

Coal gangue–slag geopolymer concrete (CGGPC) was produced with coal gangue powder calcined at 700 °C, S95 ground granulated blast-furnace slag, natural sand, crushed coarse aggregate, an alkaline activator, basalt fibers, and nano-SiO_2_. The calcined coal gangue powder and slag were adopted as the binary aluminosilicate precursors. The coal gangue powder, provided by Yunshi Calcined Coal Gangue Powder Mineral Products Processing Plant, Lingshou County, Hebei Province, China, was grey in appearance, with an average particle size of about 18 μm and a density of approximately 2.8 g/cm^3^. The slag was supplied by Hebei JieGui Mineral Products Co., Ltd., Shijiazhuang, China, and was a white powder with an average particle size of roughly 75 μm and a density of around 3.12 g/cm^3^. The oxide compositions of both precursors are presented in Table 1. According to the chemical analysis, the slag mainly consisted of CaO, SiO_2_, and Al_2_O_3_, with contents of 35.0%, 33.5%, and 17.5%, respectively, whereas the calcined coal gangue powder was dominated by SiO_2_ and Al_2_O_3_, accounting for 53.5% and 42.0%, respectively. The combination of these two precursors therefore provides both reactive Si–Al species for geopolymeric aluminosilicate networks and calcium-bearing components for the formation of calcium aluminosilicate hydrate-type gels, offering a suitable chemical foundation for the development of the coal gangue–slag geopolymer binder.

Commercially available nano-SiO_2_ powder was used as the nano-modifier. According to the supplier’s characterization data, the powder contained 99.9 wt% SiO_2_, with minor amounts of TiO_2_, Al_2_O_3_, and Fe_2_O_3_ of 0.002 wt%, 0.0031 wt%, and 0.002 wt%, respectively. The nano-SiO_2_ had a specific surface area of 220 m^2^/g and a median particle size of 20 nm. The detailed physicochemical properties of nano-SiO_2_ are presented in Table 2. Such nanoscale dimensions and high surface area may favor pore filling and provide potential sites for heterogeneous nucleation within the geopolymer matrix; however, they may also increase susceptibility to particle agglomeration when dispersion is inadequate. To improve its dispersion, nano-SiO_2_ was premixed with the mixing water and mechanically stirred before being incorporated into the fresh paste.

The alkaline activator consisted of sodium silicate solution, NaOH flakes, and additional mixing water. The original modulus of the sodium silicate solution was 3.21, which was reduced to 1.3 by adding NaOH flakes with a purity of 96%. Natural river sand, having a fineness modulus of 2.54 and an apparent density of 2635 kg/m^3^, was selected as the fine aggregate. The coarse aggregate was prepared by blending crushed stones with particle-size ranges of 5–10 mm and 10–20 mm at an equal mass ratio. The basalt fibers used in this study were 12 mm in length and approximately 17 μm in diameter, with a density of 2.69 g/cm^3^, tensile strength exceeding 2000 MPa, elastic modulus higher than 85 GPa, and elongation at break of 2.5%. The physical and mechanical properties of the basalt fibers are presented in Table 3. Owing to their high tensile capacity and stiffness, these fibers are expected to contribute to crack-bridging and toughness enhancement in geopolymer concrete.

### 2.2. Specimen Design and Mix Proportion

Based on preliminary trial tests and relevant studies, the silicate modulus of the alkaline activator, alkali dosage, water-to-binder ratio, aggregate-to-binder ratio, and sand ratio were set as 1.3, 9%, 0.41, 3.5, and 0.37, respectively. The binder system consisted of calcined coal gangue powder and slag blended at a mass ratio of 1:1. With these parameters kept constant, the nano-SiO_2_ dosage was selected as the only variable, while the basalt fiber content was fixed at 1.00%. The mixtures were designated NS0, NS0.5, NS1, NS1.5, and NS2 according to nano-SiO_2_ dosages of 0%, 0.5%, 1.0%, 1.5%, and 2.0%, respectively. The NS0 mixture, containing no nano-SiO_2_, was used as the reference mixture. The detailed mix proportions of all mixtures are presented in Table 4.

### 2.3. Preparation of Specimens

**Figure 1:**
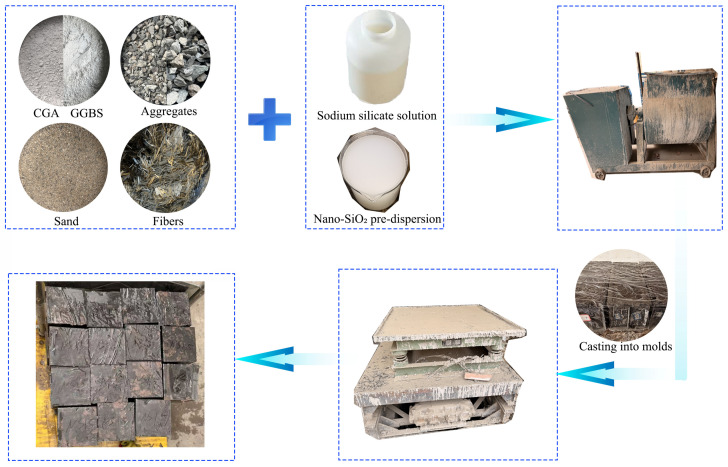
Preparation flow chart of specimens.

The specimen preparation procedure is illustrated in Figure 1. The alkaline activator was prepared 12 h prior to casting by gradually dissolving solid NaOH into sodium silicate solution under continuous stirring to obtain the target activator modulus. Meanwhile, nano-SiO_2_ was added to the mixing water and mechanically pre-dispersed to produce a relatively homogeneous suspension, thereby reducing particle agglomeration and improving its dispersion within the matrix. Calcined coal gangue powder, slag, fine aggregate, and coarse aggregate were then weighed according to the mix proportions and dry-mixed for 3 min. Basalt fibers were incorporated in two stages, with approximately 50% added during the initial dry-mixing process and the remainder gradually introduced after the addition of the alkaline activator and nano-SiO_2_ dispersion, followed by a further 3 min of mixing to minimize fiber entanglement and clustering. The fresh mixture was subsequently cast into molds and compacted on a vibrating table for 2 min, during which excess surface paste and visible air bubbles were removed to reduce casting defects. After 24 h, the specimens were demolded and cured under standard conditions at 20 ± 2 °C and a relative humidity of no less than 95% until the designated testing ages.

### 2.4. Test Methods

#### 2.4.1. Strength Tests

**Figure 2:**
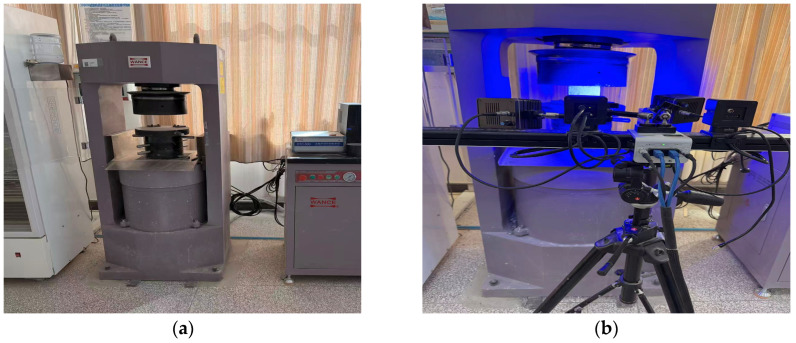
Experimental setup: (**a**) computer-controlled automatic compression testing machine; (**b**) DIC testing system.

Compressive strength and splitting tensile strength were measured at the designated curing ages in accordance with GB/T 50081–2019 [50]. All mechanical tests were carried out using a computer-controlled compression testing machine (HCT306A, Shenzhen Wance Testing Machine Co., Ltd., Shenzhen, China), which had a measurement accuracy of ±1%, as illustrated in Figure 2a. For the splitting tensile test, each cube specimen was placed in a dedicated splitting fixture, and the load was transferred through the loading device to maintain stable and aligned loading along the specified direction. For each mixture and curing age, three specimens were tested independently for compressive strength and three specimens were tested independently for splitting tensile strength. The reported compressive strength and splitting tensile strength values represent the arithmetic means of three independent specimens, and the corresponding error bars represent one standard deviation.

#### 2.4.2. Digital Image Correlation Test

Digital image correlation (DIC) was used to monitor the full-field surface displacement and strain evolution during splitting tensile loading. One specimen from each mixture was tested; therefore, the strain contours, crack-opening curves, and derived parameters represent specimen-level responses rather than group mean values.

The DIC system used in this study, as shown in Figure 2b, was supplied by XTOP 3D Technology (Shenzhen) Co., Ltd. (Shenzhen, China) and consisted of an industrial camera equipped with a 50 mm lens and a constant cold-light source. The camera was positioned approximately 500 mm from the specimen surface. A random black-on-white speckle pattern was prepared before loading. Images were acquired synchronously with the testing machine at 400 ms intervals, while load and displacement were recorded simultaneously.

The main crack was identified from the maximum principal strain field. Paired virtual points were placed on opposite sides of the crack along its local normal direction, and their relative displacement was defined as the main crack-opening displacement, w. The same point-selection criterion was applied to all mixtures. Because only one specimen per mixture was monitored, the DIC results were used for descriptive comparison rather than inferential statistical analysis.

#### 2.4.3. Mercury Intrusion Porosimetry Test

**Figure 3:**
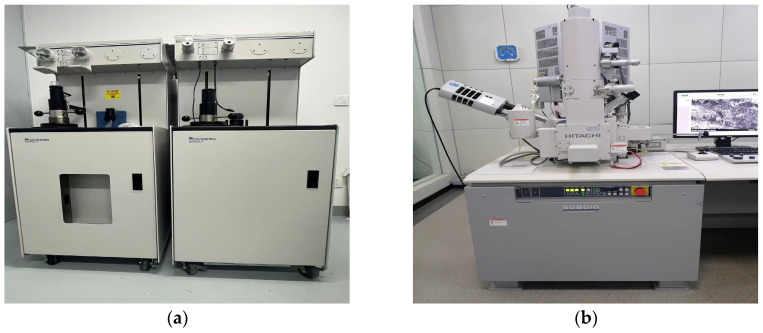
Testing equipment: (**a**) mercury intrusion porosimeter; (**b**) field-emission scanning electron microscope.

To further characterize the effect of nano-SiO_2_ on the pore structure of basalt fiber-reinforced coal gangue–slag geopolymer concrete, Mercury intrusion porosimetry (MIP) was performed on the NS0 and NS0.5 specimens using an AutoPore 9500 automatic mercury porosimeter (Micromeritics Instrument Corporation, Norcross, GA, USA), as shown in Figure 3a. The samples were collected from the internal matrix region of the specimens after 28 d of curing, while coarse aggregates, visible macrocracks, and external fracture surfaces were carefully avoided to minimize the influence of aggregates and local damage on the test results. The obtained samples were crushed into small pieces of approximately 3–5 mm, immersed in absolute ethanol for 24 h to retard further reaction, and then dried at 50 °C to constant mass. After cooling to room temperature, the dried samples were subjected to MIP testing. The pore-size distribution characteristics were obtained from the cumulative mercury intrusion volume and logarithmic differential intrusion curves, and the pore volume fractions within different pore-size ranges were calculated to evaluate the effects of nano-SiO_2_ on pore refinement, the reduction of harmful macropores, and changes in water-transport pathways.

#### 2.4.4. Water Absorption Test

To evaluate the effect of nano-SiO_2_ dosage on the water absorption behavior of basalt fiber-reinforced coal gangue–slag geopolymer concrete, cubic specimens cured for 28 d were selected for the water absorption test. Three replicate specimens were tested for each mixture, and the average value was reported as the final result. Before testing, the specimens were dried at 105 °C to constant mass and then cooled to room temperature to determine their oven-dry mass. The specimens were subsequently immersed in clean water, with the water level maintained at least 20 mm above the top surface of the specimens. At immersion durations of 1, 3, 6, 12, and 24 h, the specimens were removed from water, their surface water was wiped off, and their mass was immediately recorded. The water absorption was calculated based on the mass variation in the specimens at different immersion times, and the influence of nano-SiO_2_ dosage on pore connectivity and moisture transport capacity of the matrix was analyzed.

#### 2.4.5. Scanning Electron Microscopy Analysis

SEM specimens were collected from the regions adjacent to the main crack of the specimens after splitting tensile failure and were cut into small fragments of approximately 10–20 mm. To retard further reactions and reduce the influence of pore water on microstructural observation, the samples were first immersed in absolute ethanol for 24 h and then dried below 50 °C to constant mass. The dried samples were examined using an Apreo high-resolution field-emission scanning electron microscope (SEM; Thermo Fisher Scientific, Waltham, MA, USA), as shown in Figure 3b. The observations were conducted to characterize the changes in matrix microstructure, crack-like defects, and reaction product morphology after the incorporation of nano-SiO_2_, thereby providing a microstructural explanation for its effects on the mechanical properties and crack evolution behavior of the material.

## 3. Results and Analysis

### 3.1. Analysis of Compressive Strength Results

**Figure 4:**
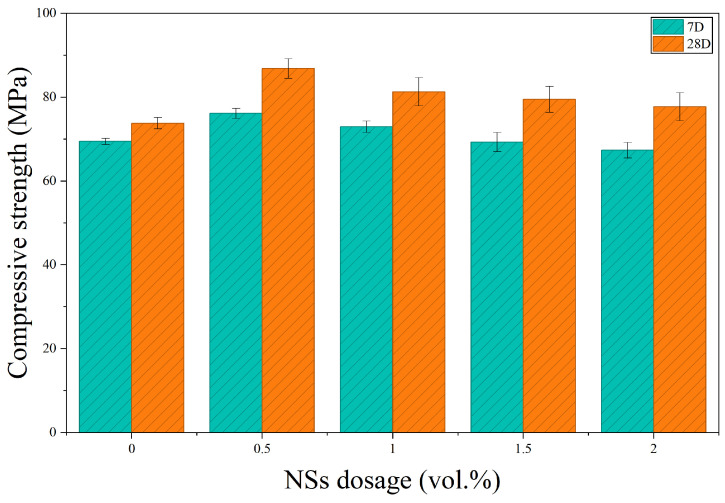
Compressive strength at different nano-SiO_2_ dosages after 7 and 28 d.

As shown in Figure 4, the compressive strength of all specimens increased with curing age, indicating that the coal gangue–slag geopolymer binder system continued to react from 7 to 28 d, leading to further densification of the matrix. The 7 d and 28 d compressive strengths of the reference mixture NS0 were 69.47 MPa and 73.77 MPa, respectively. After the incorporation of nano-SiO_2_ (NS), the compressive strength exhibited a clear dosage-dependent response. Among all mixtures, NS0.5 showed the highest strength at both 7 d and 28 d, reaching 76.13 MPa and 86.80 MPa, respectively, corresponding to increases of 9.60% and 17.67% compared with NS0. The 7 d and 28 d compressive strengths of NS1 were 72.95 MPa and 81.28 MPa, respectively, which remained higher than those of NS0. However, when the NS dosage was further increased to 1.5% and 2%, the 7 d compressive strength decreased to 69.28 MPa and 67.36 MPa, respectively, indicating that excessive NS did not continuously promote early-age strength development. At 28 d, the compressive strengths of NS1.5 and NS2 were 79.47 MPa and 77.69 MPa, respectively. Although these values were still higher than that of NS0, their enhancement was markedly lower than that of NS0.5, suggesting that the improvement in compressive strength was not linearly proportional to the NS dosage.

These results demonstrate a clear dosage-dependent strengthening response. The improvement at a relatively low nano-SiO_2_ dosage is consistent with enhanced matrix compactness and pore-structure refinement. Based on mechanisms reported in previous studies, micro-filling, heterogeneous nucleation, and enhanced reaction-product formation may have contributed to this response; however, nucleation and additional reaction-product formation were not directly verified by the present analyses. Conversely, the reduced strengthening efficiency at higher dosages may be associated with less uniform dispersion, increased water demand, particle agglomeration, or greater local heterogeneity. In terms of strength development from 7 to 28 d, the compressive strength growth rate of NS0 was only 6.19%, whereas those of the NS-modified mixtures ranged from 11.42% to 15.34%, indicating that NS contributed to continued strength development at later ages. NS0.5 showed the most pronounced strength gain, with its 28 d strength increasing by 10.67 MPa relative to its 7 d value. Overall, 0.5% nano-SiO_2_ can be regarded as the optimal dosage within the investigated range. Its strength enhancement is directly consistent with improved matrix compactness and pore refinement, whereas the possible contributions of heterogeneous nucleation and additional reaction-product formation remain mechanistic interpretations.

### 3.2. Analysis of Splitting Tensile Strength

**Figure 5:**
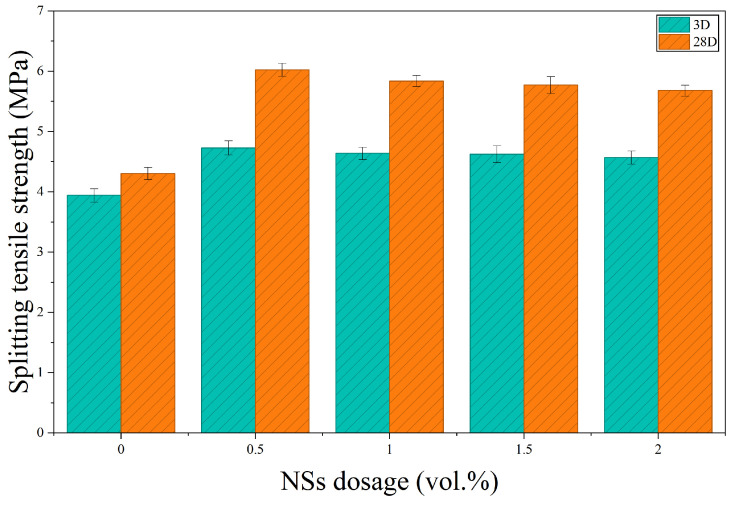
Splitting tensile strength at different nano-SiO_2_ dosages after 7 and 28 d.

As shown in Figure 5, the splitting tensile strength of all specimens increased with curing age, indicating that the coal gangue–slag geopolymer system continued to react from 7 to 28 d, leading to further refinement of the matrix structure, pore characteristics, and interfacial transition zone. The 7 d and 28 d splitting tensile strengths of the reference mixture NS0 were 3.94 MPa and 4.30 MPa, respectively. After the incorporation of nano-SiO_2_ (NS), the splitting tensile strength of all modified mixtures increased markedly at both curing ages. At 7 d, the splitting tensile strength of the NS-modified mixtures ranged from 4.57 to 4.73 MPa, representing increases of 15.91–19.97% compared with NS0, with NS0.5 showing the highest value. At 28 d, the splitting tensile strength of NS0.5 reached 6.02 MPa, corresponding to an increase of 39.97% relative to NS0. The strengths of NS1, NS1.5, and NS2 were 5.84 MPa, 5.77 MPa, and 5.68 MPa, respectively, corresponding to increases of 35.63%, 34.08%, and 31.99%. These results indicate that the improvement in splitting tensile strength was strongly dosage-dependent, with 0.5% NS providing the most effective enhancement, whereas further increases in NS dosage gradually reduced the strengthening efficiency.

Splitting tensile strength is particularly sensitive to microcracks, connected pores, and weak interfaces. The improved tensile performance at an appropriate nano-SiO_2_ dosage is therefore consistent with the observed enhancement in matrix continuity and pore refinement. Micro-filling and possible contributions from heterogeneous nucleation or enhanced gel-product formation may also be involved, as suggested by previous studies, but these processes were not directly verified in the present work. Similarly, the diminishing enhancement at higher dosages may be associated with poorer dispersion, particle agglomeration, or the development of local defects, rather than providing direct evidence for any single mechanism. In terms of strength development from 7 to 28 d, the splitting tensile strength of NS0 increased by only 9.22%, whereas that of the NS-modified mixtures increased by 24.38–27.43%, suggesting that NS not only improved early-age tensile performance but also promoted later-age strength development. In particular, the strength of NS0.5 increased from 4.73 MPa to 6.02 MPa, with a growth rate of 27.43%, indicating that 0.5% NS was the most favorable dosage for sustained matrix densification and enhancement of splitting tensile performance within the scope of this study.

### 3.3. Water Absorption

**Figure 6:**
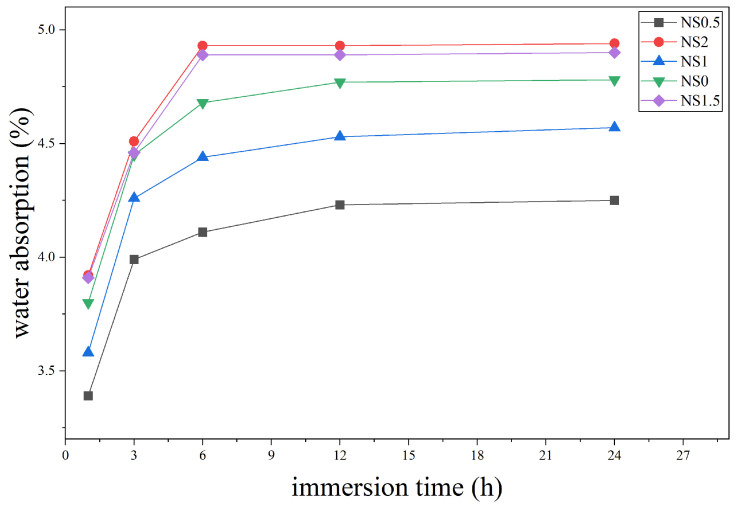
Variation in water absorption of BFCGGPC specimens with different nano-SiO_2_ dosages during 24 h immersion.

As shown in Figure 6, the water absorption of all specimens increased with immersion time, exhibiting a rapid initial increase followed by a slower growth stage. During the first 1–6 h, water absorption increased sharply, indicating that water rapidly penetrated into the specimens mainly through capillary pores and connected pores. After 6–24 h, the growth rate decreased markedly, suggesting that the internal pores were gradually filled with water and that the absorption process approached a stable state. The nano-SiO_2_ dosage had a significant effect on the water absorption behavior. The 24 h water absorption of the NS0 mixture was approximately 4.78%, whereas the NS0.5 mixture exhibited the lowest water absorption throughout the immersion period, with a 24 h value of approximately 4.25%, representing a reduction of about 11.1% compared with NS0. The lower water absorption of the NS0.5 mixture is consistent with improved matrix compactness, a reduction in connected pores, and refinement of the pore-size distribution. Micro-filling may have contributed to these changes, while enhanced gel formation remains a plausible interpretation rather than a process directly verified by the present measurements.

With a further increase in nano-SiO_2_ dosage, the water absorption increased again. The NS1 mixture still showed lower water absorption than NS0 but higher water absorption than NS0.5, indicating that its pore-refinement effect was weakened. When the dosage increased to 1.5% and 2%, the water absorption increased markedly and exceeded that of NS0; in particular, the NS2 mixture showed the highest 24 h water absorption, approximately 4.93%. The increased water absorption at higher nano-SiO_2_ dosages may reflect a greater prevalence of micropores, microcracks, or connected water-transport pathways. Poorer nanoparticle dispersion, particle agglomeration, and increased local heterogeneity represent possible explanations for this response; however, these mechanisms were not directly characterized in the present study. Overall, water absorption exhibited a non-monotonic trend with increasing nano-SiO_2_ dosage, first decreasing and then increasing. The NS0.5 mixture showed the best performance, indicating that 0.5% nano-SiO_2_ was the optimal dosage within the scope of this study for refining the pore structure, improving matrix compactness, and reducing water ingress.

### 3.4. DIC-Based Analysis of Splitting Tensile Failure Evolution

#### 3.4.1. Evolution of Maximum Principal Strain During Splitting Tensile Loading

**Figure 7:**
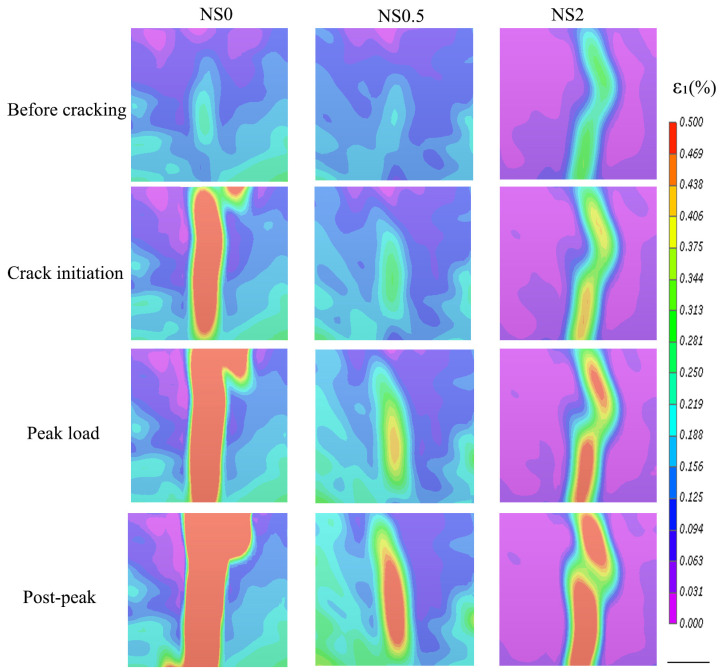
Maximum principal strain fields during splitting tensile loading at different nano-SiO_2_ dosages.

As shown in Figure 7, the DIC-derived maximum principal strain contours provide a direct visualization of damage evolution and crack propagation during the splitting tensile process. Before macroscopic cracking, local strain concentration had already appeared in the central splitting region of the NS0 specimen. In contrast, the NS0.5 specimen exhibited a more uniform strain distribution without the formation of a continuous high-strain band, indicating that 0.5% nano-SiO_2_ delayed the initiation of tensile damage. For the NS2 specimen, however, a curved strain-localization band was observed, suggesting that excessive nano-SiO_2_ may introduce local heterogeneity and promote premature damage localization. As loading proceeded, a continuous high-strain band rapidly formed and propagated along the loading diameter in the NS0 specimen, almost penetrating the specimen height at the peak-load stage, which is characteristic of brittle splitting failure. By comparison, the NS0.5 specimen exhibited only limited and discontinuous strain concentration, and the penetration of the main crack was markedly delayed. In the NS2 specimen, several interconnected high-strain regions developed along a curved weakened path, indicating that although the crack path became more tortuous, local damage evolved earlier.

The quantitative results in Table 5 further confirm these observations. The maximum principal tensile strain and line-averaged principal tensile strain of the NS0 specimen were both substantially higher than those of the NS-modified specimens, indicating more severe tensile deformation and damage accumulation during splitting failure. For NS0.5, the maximum principal tensile strain decreased to 0.475–0.827%, while the line-averaged principal tensile strain was only 0.112–0.134%, demonstrating that 0.5% nano-SiO_2_ significantly reduced the overall tensile deformation level and suppressed main-crack propagation. Although the strain-localization coefficient of NS0.5 was slightly higher than that of NS0, this resulted from the lower background strain in the non-cracked region, which made the high-strain zone more concentrated, rather than indicating more severe damage. The NS2 specimen showed a lower maximum principal tensile strain than NS0, but its line-averaged principal tensile strain was higher than that of NS0.5, and its strain-localization coefficient was lower, indicating that excessive nano-SiO_2_ caused tensile deformation to accumulate more diffusely along the curved weakened path. Overall, 0.5% nano-SiO_2_ was most effective in reducing tensile strain, restricting the strain-localization zone, and delaying main-crack penetration. The less favorable response of the NS2 mixture is consistent with increased local heterogeneity; nanoparticle agglomeration represents one possible explanation but was not directly observed by DIC.

#### 3.4.2. Evolution of Main Crack Opening Displacement

**Figure 8:**
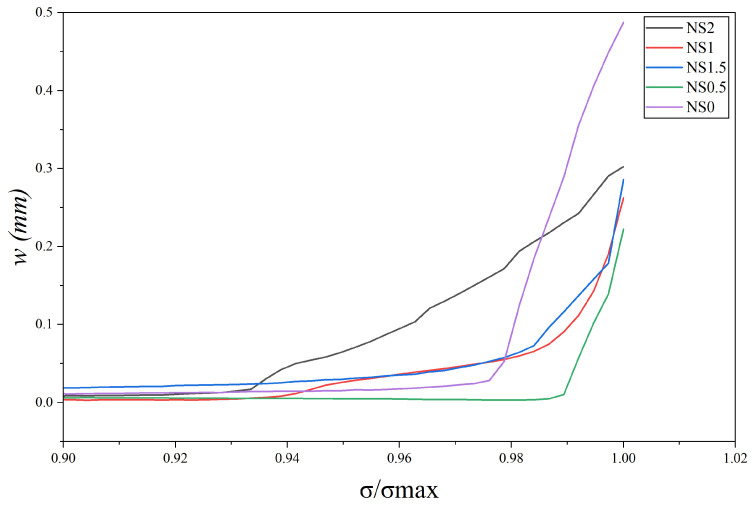
Evolution curves of the main crack opening displacement of specimens with different nano-SiO_2_ dosages.

To quantitatively evaluate the opening behavior of the main crack during splitting tensile loading, the crack width (*W*) at different normalized stress levels was extracted from the DIC displacement field, and the key crack-opening parameters were calculated, as shown in Figure 8 and Table 6. Overall, the crack width of all specimens increased with increasing $\sigma /\sigma_{\max}$. However, clear differences were observed among mixtures with different nano-SiO_2_ dosages in terms of crack-opening initiation, pre-peak crack growth rate, and peak crack width.

For the NS0 specimen, the crack width remained relatively small during most of the pre-peak loading stage. At $\sigma /\sigma_{\max}=0.98$, the crack width was 0.087 mm. However, as the load further approached the peak value, the crack width increased rapidly and reached 0.487 mm at peak load. The normalized stress level corresponding to the threshold crack width $W_{\mathrm{th}}=0.05$ mm was 0.978, indicating that the main crack in the NS0 specimen had already entered a pronounced opening stage before peak load. This result suggests that, once initiated, the crack in the reference specimen propagated rapidly under splitting tensile loading, with a sharp increase in crack opening near the peak load, indicating a pronounced brittle splitting failure mode.

Compared with the NS0 specimen, the NS0.5 specimen exhibited the most effective crack-opening suppression. At $\sigma /\sigma_{\max}=0.98$, the crack width of NS0.5 was only 0.003 mm, much lower than that of NS0, indicating that the main crack had not yet undergone obvious opening before peak load. Meanwhile, the normalized stress level at which NS0.5 reached $W_{\mathrm{th}}$ was 0.992, the highest among all mixtures, demonstrating that 0.5% nano-SiO_2_ effectively delayed the transition of the crack into the rapid-opening stage. At peak load, the crack width of NS0.5 was 0.222 mm, representing a 54.4% reduction relative to NS0 and the lowest value among all groups. These results indicate that an appropriate nano-SiO_2_ dosage can markedly reduce crack opening at the peak stage and improve the resistance of the specimen to crack widening, which is consistent with its higher splitting tensile strength and delayed localization of maximum principal strain observed by DIC.

The crack-width evolution of the NS1 and NS1.5 specimens fell between those of NS0.5 and NS2. At $\sigma /\sigma_{\max}=0.98$, the crack widths of NS1 and NS1.5 were 0.058 mm and 0.061 mm, respectively, both lower than that of NS0 but clearly higher than that of NS0.5. The normalized stress level corresponding to $W_{\mathrm{th}}$ was 0.974 for both mixtures, indicating that obvious crack opening occurred earlier than in NS0.5. At peak load, the crack widths of NS1 and NS1.5 were 0.262 mm and 0.286 mm, corresponding to reductions of 46.1% and 41.3% relative to NS0, respectively. These results show that 1% and 1.5% nano-SiO_2_ still limited crack opening to some extent, but their crack-suppression efficiency was lower than that of the 0.5% dosage.

The NS2 specimen showed clear characteristics of early crack opening. The normalized stress level corresponding to $W_{\mathrm{th}}$ was only 0.941, the lowest among all mixtures, indicating that excessive nano-SiO_2_ caused the crack to enter the obvious opening stage earlier. At $\sigma /\sigma_{\max}=0.98$, the crack width of NS2 had reached 0.182 mm, substantially higher than those of the other nano-SiO_2_-modified specimens, indicating more pronounced pre-peak crack propagation. Although the peak crack width of NS2 was 0.303 mm, still lower than that of NS0, its earlier crack-opening initiation and larger pre-peak crack width indicate that excessive nano-SiO_2_ did not further improve crack-control capacity. This may be associated with nanoparticle agglomeration, increased local heterogeneity, and the formation of weak interfacial regions, which could promote premature pre-peak damage development.

The crack-width curves and quantitative parameters indicate that nano-SiO_2_ exerted a distinct non-monotonic effect on crack-width evolution. As the nano-SiO_2_ dosage increased from 0.5% to 2%, the peak crack width gradually increased from 0.222 mm to 0.303 mm, while the normalized stress level corresponding to the crack-opening threshold decreased from 0.992 to 0.941, demonstrating that excessive nano-SiO_2_ weakened the crack-opening suppression effect. The NS0.5 specimen simultaneously exhibited the lowest W_0.98_, the lowest $W_{\mathrm{peak}}$, and the highest $(\sigma /\sigma_{max})\text{·}W=W_{th}$, confirming that 0.5% nano-SiO_2_ was the most effective dosage for delaying crack opening and reducing the peak crack width. This crack-width evolution agrees well with the splitting tensile strength, maximum principal strain contours, and line-extracted strain results, further confirming that an appropriate nano-SiO_2_ dosage improves stable crack propagation resistance in basalt fiber-reinforced geopolymer concrete by enhancing matrix compactness and the fiber–matrix interfacial interaction.

### 3.5. Microstructural Mechanism Analysis

#### 3.5.1. SEM Micro-Mechanism Analysis

**Figure 9:**
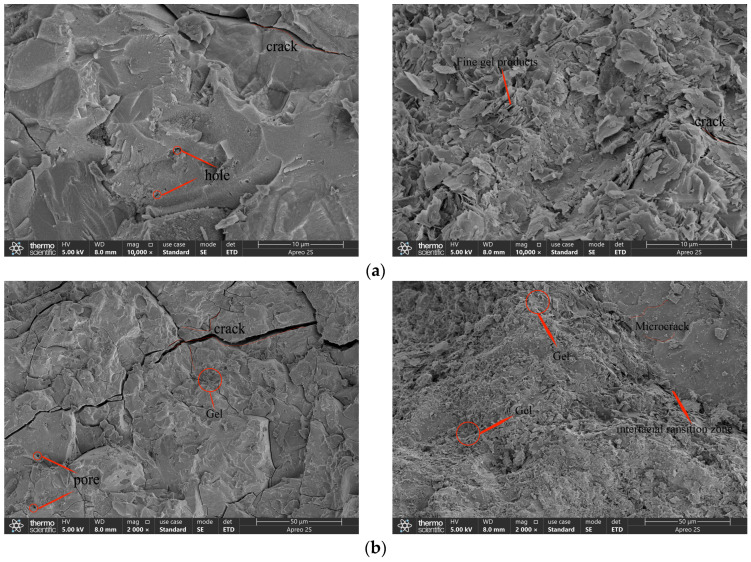
SEM images of typical fracture surfaces: (**a**) NS0; (**b**) NS0.5.

To clarify the microstructural origin of the reduced water absorption and improved crack-control capacity of the NS0.5 mixture, SEM observations were conducted on the fractured matrix regions of the NS0 and NS0.5 specimens. As shown in Figure 9, the results reveal clear differences in matrix compactness, crack-like defects, and the morphology of reaction products between the two mixtures. The fracture surface of the NS0 specimen was relatively loose and discontinuous, with numerous open cracks, pores, and local interfacial gaps. These defects indicate that the matrix without nano-SiO_2_ contained more coarse pores and connected weak zones, which could readily form water transport pathways and induce local stress concentration and rapid crack propagation during splitting tensile loading. This observation is consistent with the higher water absorption of the NS0 specimen and the rapid increase in crack width identified by DIC analysis.

In contrast, the fracture surface of the NS0.5 specimen was denser and was covered with more flocculent, lamellar, and gel-like reaction products, while large open cracks and coarse pores were substantially reduced. This indicates that 0.5% nano-SiO_2_ promoted the formation of geopolymeric gel products through micro-filling and nucleation effects, thereby filling local pores and improving the continuity of the matrix. It should be noted that individual nano-SiO_2_ particles cannot be directly and accurately identified in the hardened matrix from SEM images, because they may have participated in the alkaline reaction, been encapsulated by aluminosilicate gels, or become indistinguishable from gel products due to their nanoscale size. Therefore, the SEM results in this study are mainly used as morphological evidence for matrix densification, pore-structure refinement, and the reduction of crack-like defects, rather than as direct proof of the presence of individual nano-SiO_2_ particles. Overall, 0.5% nano-SiO_2_ improved the microstructural integrity of the basalt fiber-reinforced geopolymer concrete, providing a microstructural explanation for the lower water absorption, smaller crack width, delayed tensile strain localization, and higher splitting tensile strength of the NS0.5 mixture.

#### 3.5.2. Mercury Intrusion Porosimetry Analysis

**Figure 10:**
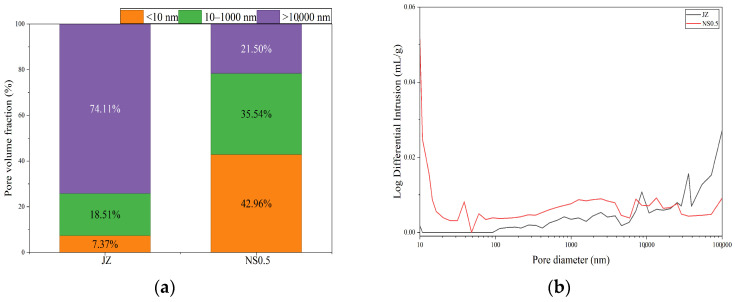
MIP-derived pore-structure characteristics: (**a**) pore-size fractions; (**b**) differential intrusion curves.

Since the NS0.5 mixture exhibited the best overall performance in terms of mechanical properties, water absorption, and crack control, the NS0 and NS0.5 mixtures were selected for comparative MIP analysis to elucidate the pore-structure mechanism associated with an appropriate NS dosage. Figure 10a presents the pore volume fractions within different pore-size ranges, while Figure 10b shows the logarithmic differential mercury intrusion curves of the two mixtures. The results indicate that the incorporation of 0.5% nano-SiO_2_ markedly altered the pore-size distribution, shifting the pore structure from a macropore-dominated system to one dominated by fine pores and gel pores.

As shown in Figure 10a, pores larger than 10,000 nm accounted for 74.11% of the pore volume in the NS0 mixture, whereas pores smaller than 10 nm accounted for only 7.37%. This indicates that the mercury-accessible pore volume in NS0 was mainly governed by macropores, crack-like pores, or connected interfacial pores. Although such pores may not necessarily increase the total number of pores, they can readily form rapid water transport pathways and reduce the resistance of the matrix to crack propagation. In contrast, the pore volume fraction of pores larger than 10,000 nm in NS0.5 decreased to 21.50%, while that of pores smaller than 10 nm increased significantly to 42.96%. Meanwhile, the pore volume fraction in the range of 10–10,000 nm increased from 18.51% in NS0 to 35.54% in NS0.5. These results demonstrate that 0.5% nano-SiO_2_ effectively reduced the proportion of coarse connected pores and crack-like pores, while shifting the pore-size distribution toward smaller pore ranges.

The logarithmic differential intrusion curves in Figure 10b further confirm the pore-refinement effect. The NS0 mixture showed a relatively low mercury intrusion contribution in the small-pore region, whereas the intrusion contribution increased gradually in the macropore range of 10,000–1,000,000 nm, indicating that its pore volume was mainly derived from larger pore channels. By contrast, the NS0.5 mixture exhibited a distinct peak near 10 nm, suggesting a marked increase in fine pores and gel pores. At the same time, the differential intrusion volume of NS0.5 in the macropore region was lower than that of NS0, indicating that coarse connected pores were effectively reduced. This shift in pore-size distribution suggests that an appropriate dosage of nano-SiO_2_ promotes geopolymer gel formation through filling and nucleation effects, fills original pores, refines pore throats, increases pore tortuosity, and thereby reduces water migration capacity.

It is worth noting that the total porosity of NS0.5 decreased by approximately 63.9% compared with NS0, whereas its 24 h water absorption decreased by only approximately 11.1%; these two reductions were not proportional. This discrepancy is mainly attributable to the different objects characterized by the two tests. The MIP specimens were taken from the matrix region while avoiding coarse aggregates and macroscopic defects, and therefore mainly reflect the pore structure at the paste scale. In contrast, the water absorption test was conducted on whole concrete specimens, in which the limited water absorption capacity of aggregates, the interfacial transition zones, surface defects introduced during casting, and specimen-scale heterogeneity may dilute the relative contribution of matrix improvement. In addition, water ingress within 24 h is mainly controlled by connected capillary pores and macropores. Although the increased fraction of fine pores and gel pores below 10 nm in NS0.5 contributes to the total pore volume, these pores make a limited contribution to short-term water transport. Therefore, the reduction in water absorption of NS0.5 should be understood as the combined result of reduced total porosity and refined pore-size distribution. In particular, the absolute volume of coarse connected pores larger than 10,000 nm decreased sharply from approximately 10.3% (13.93% × 74.11%) to approximately 1.1% (5.03% × 21.50%), which was the dominant factor inhibiting water transport.

Overall, the MIP results demonstrate that 0.5% nano-SiO_2_ significantly optimized the pore structure of basalt fiber-reinforced geopolymer concrete in two aspects: first, it reduced the total porosity from 13.93% to 5.03%; second, it transformed the pore structure from macropore-dominated to fine-pore- and gel-pore-dominated, with a substantial reduction in the absolute volume of coarse connected pores and crack-like pores. These results are consistent with the lowest water absorption, the smallest DIC-measured crack width, and the highest splitting tensile performance of the NS0.5 mixture, indicating that an appropriate nano-SiO_2_ dosage synergistically enhanced the resistance to water ingress and crack-control capacity through the dual mechanisms of porosity reduction and pore-size refinement.

## 4. Discussion

Based on the combined results of mechanical properties, water absorption, DIC-based crack evolution, and microstructural pore characterization, the modification effect of nano-SiO_2_ on basalt fiber-reinforced coal gangue–slag geopolymer concrete can be interpreted as a multiscale response involving matrix densification, pore-structure refinement, and crack-growth suppression. At a nano-SiO_2_ dosage of 0.5%, all measured properties reached their optimum values. The SEM observations showed a denser fracture morphology with fewer coarse pores and crack-like defects, while the MIP results directly demonstrated a substantial reduction in total porosity and a shift in pore-size distribution toward finer pore ranges. These findings provide direct evidence of matrix densification and pore refinement. Micro-filling may have contributed to these changes, whereas heterogeneous nucleation and additional gel formation remain plausible interpretations rather than directly verified processes.

The improvement in splitting tensile strength induced by nano-SiO_2_ was greater than that in compressive strength, suggesting that nano-SiO_2_ is more effective in mitigating tensile-governed defects. An appropriate nano-SiO_2_ dosage can improve matrix continuity and fiber–matrix interfacial bonding, thereby reducing the local stress concentration required for crack initiation and enabling the bridging, pull-out, and energy-dissipation effects of basalt fibers to be more fully mobilized. Accordingly, the NS0.5 mixture not only exhibited the highest splitting tensile strength, but also showed delayed strain localization, reduced peak crack width, and postponed main-crack penetration in the DIC results. Therefore, the lower water absorption, smaller crack width, and higher tensile strength of the NS0.5 mixture are not isolated phenomena, but are collectively attributed to matrix densification and pore-structure optimization.

However, the enhancement effect of nano-SiO_2_ exhibited a pronounced non-monotonic dependence on dosage. Although some mechanical indicators of the NS1, NS1.5, and NS2 mixtures remained higher than those of the NS0 mixture, their enhancement efficiency, water-absorption reduction, and crack-opening control were all inferior to those of the NS0.5 mixture. The inferior performance of the higher-dosage mixtures may reflect the formation of local weak zones, micropores, or interfacial defects. Poorer nanoparticle dispersion, increased water demand, particle agglomeration, and greater local heterogeneity represent possible explanations for this response; however, these factors were not directly characterized in the present study. The curved weakened path and earlier crack opening observed in the DIC strain field of the NS2 mixture therefore indicate greater susceptibility to local defects, rather than directly verifying nanoparticle agglomeration.

Taken together, the mechanical, DIC, SEM, and MIP results support a multiscale interpretation in which the directly observed matrix densification and pore-structure refinement are associated with improved strength, reduced water ingress, and delayed crack evolution. Micro-filling is consistent with the nanoscale particle dimensions and the measured reduction in coarse pore volume. Heterogeneous nucleation and additional gel formation may also have contributed to the observed changes, as suggested by previous studies and the gel-like morphology observed by SEM; however, these processes were not directly verified by the present SEM and MIP measurements. Similarly, the reduced performance at higher nano-SiO_2_ dosages may reflect poorer dispersion, increased water demand, particle agglomeration, or greater local heterogeneity, but the present experiments do not isolate these individual causes. The proposed interpretation should therefore be regarded as a plausible mechanism consistent with the combined observations rather than as a fully resolved causal model.

From the perspective of material design, the crack resistance of coal gangue–slag geopolymer concrete cannot rely solely on fiber reinforcement, nor can it be achieved simply by increasing the nano-SiO_2_ dosage. A more rational strategy is to ensure the effective dispersion of nanoparticles so that they preferentially refine the pore structure and strengthen the interface, while working together with the crack-bridging and toughening effects of basalt fibers to improve crack-control capacity. This study demonstrates that 0.5% nano-SiO_2_ provides a favorable balance among strength enhancement, reduced water absorption, and crack suppression. Nevertheless, because the basalt fiber dosage was fixed and the SEM and MIP analyses mainly compared the NS0 and NS0.5 mixtures, the fiber–nanoparticle interaction and the possible defect-formation mechanisms associated with excessive nano-SiO_2_ require further investigation using direct characterization of nanoparticle dispersion and reaction products.

## 5. Conclusions

This study clarifies the coupled effects of nano-SiO_2_ dosage on matrix densification, tensile-damage localization, crack opening, and water transport in basalt fiber-reinforced coal gangue–slag geopolymer concrete. The principal scientific findings and engineering implications are summarized as follows:

(1) The effect of nano-SiO_2_ was distinctly non-monotonic and was governed by the balance between its microstructural activity and dispersion state. Within the investigated dosage range, 0.5% nano-SiO_2_ provided the most favorable overall response. The improvement was more pronounced in splitting tensile strength than in compressive strength, indicating that nano-SiO_2_ primarily enhanced defect-sensitive tensile performance by improving matrix continuity rather than merely increasing bulk compressive capacity. Further increases in dosage produced diminishing benefits, which may be associated with less uniform dispersion, increased water demand, particle agglomeration, or greater local heterogeneity. Because these factors were not directly measured, they are proposed as possible explanations rather than established causes.
(2) The main crack-control contribution of 0.5% nano-SiO_2_ was not limited to increasing the peak load. DIC measurements showed that it delayed maximum principal strain localization, restricted the development of a continuous high-strain band, and postponed main-crack penetration during splitting tensile loading. The peak crack-opening displacement decreased by 54.4% relative to NS0. These results demonstrate that an appropriate nano-SiO_2_ dosage alters the tensile failure process from rapid localized crack opening toward a more delayed and stable damage-evolution mode.
(3) The macroscopic mechanical and cracking responses were associated with substantial pore-structure refinement. The addition of 0.5% nano-SiO_2_ reduced the total porosity from 13.93% to 5.03%, markedly decreased the proportion of coarse connected and crack-like pores, and increased the fraction of fine pores. The SEM and MIP evidence therefore directly supports matrix densification and pore refinement. These observations are consistent with a possible contribution from micro-filling and may also be compatible with heterogeneous nucleation and additional gel formation; however, the latter mechanisms were not directly verified in this study. The refined and more continuous matrix provides a plausible explanation for the concurrent improvements in tensile strength, crack resistance, and resistance to water ingress.
(4) From an engineering perspective, a low and well-dispersed nano-SiO_2_ dosage is more effective than simply increasing the nanoparticle content. Under the materials, mixture proportions, and curing conditions adopted in this study, 0.5% nano-SiO_2_ provides a practical mixture-design reference for basalt fiber-reinforced coal gangue–slag geopolymer concrete used in applications where tensile cracking and water ingress are important performance constraints. However, this value should not be treated as a universal optimum. Engineering application requires control of nanoparticle dispersion, mixing sequence, water demand, and workability. Further studies should verify long-term durability, larger-scale structural performance, and the interaction between nano-SiO_2_ dosage and basalt fiber content.

## Figures and Tables

**Table 1:** Supplier-reported oxide compositions (wt%) and densities of the cementitious precursors.

Materials	CaO	SiO_2_	Al_2_O_3_	SO_3_	Fe_2_O_3_	MgO	Balance *	Density
slag	35	33.5	17.5	1.65	1.03	6.01	5.31	3.12
coal gangue	0.15	53.5	42	-	1.8	-	2.55	2.8

* The balance was obtained by subtracting the reported major oxide contents from 100%, and may comprise trace constituents and/or loss on ignition (LOI) according to the supplier’s composition data.

**Table 2:** Physicochemical properties of nano-SiO_2_ (mass%).

Property	SiO_2_	TiO_2_	Al_2_O_3_	Fe_2_O_3_	Balance	Specific Surface Area (m^2^/g)	Size (nm)
Value	99.9	0.002	0.0031	0.002	0.0929	220	20

**Table 3:** Manufacturer-reported physical and mechanical properties of basalt fiber (BF).

Length (mm)	Fiber Density (g/cm^3^)	Tensile Strength (MPa)	Tensile Elastic Modulus (GPa)	Diameter/(μm)	Elongation at Break (%)
12	2.69	>2000	>85	17	2.5

**Table 4:** Mix proportions of BFCGGPC (kg/m^3^).

Test Group	CG	Slag	Water Glass	NaOH	Water	Sand	Coarse Aggregate	Fiber Dosage (vol.%)	NS Dosage (vol.%)
NS0	239	239	194	34	73	618	1053	1	0
NS0.5	239	239	194	34	73	618	1053	1	0.5
NS1	239	239	194	34	73	618	1053	1	1
NS1.5	239	239	194	34	73	618	1053	1	1.5
NS2	239	239	194	34	73	618	1053	1	2

**Table 5:** Strain-localization parameters extracted from the maximum principal strain field at different transect positions.

Group	Line Position	$\varepsilon_{1,\max}^{L}$	${\bar{\varepsilon }}_{1}^{L}$	$K_{L}$	${\bar{K}}_{L}\pm SD$
NS0	0.25	1.993	0.463	4.307	
	0.5	1.868	0.394	4.745	4.874 ± 0.643
	0.75	2.473	0.444	5.572	
NS0.5	0.25	0.475	0.128	3.705	
	0.5	0.827	0.134	6.161	5.24 ± 1.338
	0.75	0.658	0.112	5.855	
NS2	0.25	0.502	0.188	2.677	
	0.5	0.739	0.233	3.168	2.77 ± 0.36
	0.75	0.639	0.259	2.467	

Note: $\varepsilon_{1,\max}^{L}$ denotes the maximum value of the principal tensile strain extracted along a given transect line, while ${\bar{\varepsilon }}_{1}^{L}$ represents the corresponding line-averaged principal strain. The strain-localization coefficient is calculated as $K_{L}=\varepsilon_{1,\max}^{L}/{\bar{\varepsilon }}_{1}^{L}$. The reported ${\bar{K}}_{L}\pm SD$ gives the mean value and spatial dispersion of $K_{L}$ obtained from the three horizontal transects within one specimen; therefore, it reflects intra-specimen spatial variation rather than the scatter among replicated specimens.

**Table 6:** Main crack-opening parameters of specimens with different nano-SiO_2_ dosages.

Group	$W_{0.98}$	$W_{Peak}$	$W_{th}$ (mm)	Reduction Rate of $w_{p}$ Relative to NS0 (%)
NS0	0.087	0.487	0.978	0
NS0.5	0.003	0.222	0.992	54.4
NS1	0.058	0.262	0.974	46.1
NS1.5	0.061	0.286	0.974	41.3
NS2	0.182	0.303	0.941	37.9

Note: $W_{0.98}$ denotes the crack width at the normalized stress level of σ/σ_max = 0.98; $W_{peak}$ denotes the crack width corresponding to the peak load; $W_{th}$ is the predefined crack-opening threshold, which was set to 0.05 mm in this study; $(\sigma /\sigma max)\cdot W=W_{th}$ represents the normalized stress level at which the crack width reaches $W_{th}$. The reduction rate was calculated based on the peak crack width of each group relative to that of the NS0 group. When the target value was not directly available from the original data points, linear interpolation was used.

## Data Availability

The original contributions presented in this study are included in the article. Further inquiries can be directed to the corresponding author.
